# A Work in Progress: National Opioid Prescription Reductions Across Orthopaedic Subspecialties in a Contemporary Medicare Sample of 5,026,911 Claims

**DOI:** 10.5435/JAAOSGlobal-D-21-00080

**Published:** 2021-05-20

**Authors:** Alexander J. Acuña, Tarun K. Jella, Linsen T. Samuel, Thomas B. Cwalina, Todd S. Kim, Atul F. Kamath

**Affiliations:** From the Department of Orthopaedic Surgery, Cleveland Clinic Foundation, Cleveland, OH (Acuña, Jella, Dr. Samuel, Cwalina, Dr. Kamath), and the Department of Orthopaedic Surgery, Sutter Health–Burlingame Center, Burlingame, CA (Dr. Kim).

## Abstract

As the opioid epidemic continues in the United States, efforts by orthopaedic surgeons to reduce opioid prescriptions remain critical. Although previous studies have demonstrated reductions in prescriptions across surgical specialties, there is limited information regarding contemporary trends in opioid prescriptions across orthopaedic subspecialties. Our analysis sought to estimate the frequency and trends of opioid prescriptions among Medicare Part D enrollees.

**Methods:**

The Medicare Provider Utilization and Payment Data: Part D Prescriber Public Use Files from Centers of Medicare and Medicare from 2014 to 2018 were analyzed. These data were merged with the National Provider Identifier Registry to identify the subspecialty of providers. Prescriber opioid prescription rate, days per claim, and claims per patient were calculated. Temporal trends were tested using linear regression. Poisson regression was used to calculate annual adjusted incidence rate ratios while controlling for year, surgeon sex, average patient comorbidity risk score, and average patient age.

**Results:**

We analyzed 5,026,911 opioid claims prescribed to 2,661,762 beneficiaries. Among all orthopaedic surgeons, the opioid prescription rate per 100 beneficiaries significantly decreased over the study period from 52.99 (95% CI, 52.6 to 53.37) to 44.50 (44.06 to 44.93) (*P* = 0.002). This decrease was observed for each subspecialty (all *P* values < 0.05). Similar significant reductions were appreciated across cohorts in the number of claims per beneficiary (all *P* values < 0.05). The opioid prescription rate among all orthopaedic surgeons and each subspecialty decreased significantly over the study period after controlling for various patient and surgeon characteristics (all *P* values < 0.05).

**Conclusion:**

Orthopaedic surgeons across subspecialties have reduced their rates of opioid prescriptions over recent years. Although increased prescription-limiting legislation, alternative methods of pain control, and prescriber reeducation regarding the correct quantity of opioids needed for postoperative pain relief, ongoing research, and efforts are needed to translate these reductions into clinically meaningful changes.

As the number of deaths attributed to the opioid epidemic continues to rise in the United States,^1^ there remain ongoing efforts among healthcare systems and governing agencies to limit the overprescription of opioid analgesics by healthcare providers.^2^ Given that orthopaedic surgeons have historically been identified as some of the highest prescribers of opioid medications,^3^ the appropriate use of these medications for musculoskeletal pain relief has been extensively evaluated.^2^ Despite these efforts and studies across orthopaedic subspecialties demonstrating adverse outcomes associated with perioperative opioid use, large quantities of opioids continue to be frequently prescribed for patients undergoing orthopaedic procedures.^4,5^

Although legislation targeting overprescription, prescription drug monitoring programs, and alternative pain alleviation strategies have emerged over recent years, it is unclear how this has affected the amount of opioid medication prescribed by orthopaedic surgeons. Analyses evaluating changes in opioid prescribing patterns over time have primarily been limited to other medical and surgical subspecialties,^6,7^ with significant limitations demonstrated among studies exploring these temporal trends in orthopaedic surgery. Notably, although Romman et al^8^ demonstrated a 16% reduction in opioid claims among providers classified under orthopaedic surgery between 2013 and 2017, these authors failed to include orthopaedic hand surgeons, did not stratify by subspecialty, and did not report any statistics in their analysis. Therefore, given the limitations in the current literature, more comprehensive analyses of how opioid prescription habits among orthopaedic surgeons have changed over contemporary time frames are needed.

As part of the mitigation efforts to control the opioid epidemic, there have been continual efforts to reduce opioid prescriptions given by orthopaedic surgeons. Although previous studies have demonstrated reductions in prescriptions across surgical specialties, there is a paucity of information regarding contemporary trends in opioid prescriptions across orthopaedic subspecialties. This information could help the American Academy of Orthopaedic Surgeons (AAOS), as well as related policymakers and leadership societies, recognize the efficacy of previous efforts to subsequently improve future reduction strategies. Therefore, our analysis sought to evaluate the frequency and trends of opioid prescriptions provided by orthopaedic surgeons to Medicare Part D enrollees between 2014 and 2018. In addition, we aimed to evaluate these trends by the subspecialty of the prescribing orthopaedic surgeon.

## Methods

### Databases

The Medicare Provider Utilization and Payment Data: Part D Prescriber Public Use File (PUF) provides information on prescription drugs prescribed by physicians and healthcare providers paid through the Medicare Part D Prescription Drug Program. This data set contains individual provider-level information, such as sex, practice location, and total prescription claim count. In addition, data regarding the Medicare patient population of each provider are available, including average patient age and mean Centers for Medicare and Medicaid hierarchical condition category comorbidity risk score. The data set additionally includes opioid-specific prescription data, such as opioid claim counts, opioid day supply, and the number of patients receiving opioids from each provider registered for Medicare Part D. According to the PUF methodology files, each included claim represents either an original opioid prescription or a refill.^9^

These data were merged with the publicly available National Plan and Provider Enumeration System of the Centers for Medicare and Medicaid. This registry organizes all healthcare providers who are currently practicing by their National Provider Identifier (NPI), a unique identification number assigned to any Health Insurance Portability and Accountability Act covered entity. This includes healthcare organizations and individual providers. Information available from the NPI registry includes the sex and practice location of providers, as well as their discrete subspecialty taxonomy codes.^10^ All included information is self-reported by providers when applying for this NPI number.

### Data Selection

The Part D Prescriber PUFs from 2014 to 2018 were queried for all providers whose specialty was listed as one of the following: Orthopaedic Surgery, Orthopedic Surgery, or Hand Surgery. The subsequent list of surgeons was verified using taxonomy code 207X00000X—Orthopaedic Surgery (eg, plastic surgeons who practice hand surgery were excluded). We then further stratified surgeons based on taxonomy codes pertaining to orthopaedic subspecialties. Specifically, the following codes were identified: 207XS0114X—Adult Reconstruction, 207XX0004X—Foot and Ankle, 207XS0106X—Hand Surgery, 207XX0801Z—Orthopaedic Trauma, 207XS0117X—Spine Surgery, and 207XX0005X—Sports Medicine. The taxonomy codes for surgeons without these specific taxonomy codes were then manually reviewed by two authors (A.J.A. and T.K.J.) and assigned to an orthopaedic subspecialty if certain taxonomy classifications were deemed appropriate. For example, surgeons with the taxonomy code 2086S0105X (Surgery of the Hand) were allocated to the Hand Surgery cohort, whereas those with taxonomy code 2086S0127X (Trauma Surgery) were assigned to the Orthopaedic Trauma cohort. Surgeons with taxonomy codes pertaining to other medical specialties and other healthcare professions (including registered nurses, physician assistants, chiropractors, and podiatrists) were excluded. We similarly excluded any surgeons with the taxonomy code 390200000X—Student in an Organized Health Care Education/Training Program.

### Statistical Analysis

Univariate statistics were used to describe orthopaedic surgeon cohorts both across years and across specialties. For the primary analysis, prescriber opioid prescription rate, days per claim, and claims per patient were calculated. We calculated an opioid prescription rate (opioid claim count/total claim count), days per claim (opioid day supply/opioid claim count), and claims per patient (opioid claim count/opioid patient count). All annual prescriber averages for each of the three values were calculated over the period. A histogram was created to evaluate how the distribution of opioid prescription rates among orthopaedic surgeons changed from 2014 to 2018. In addition, all data were stratified to calculate yearly averages for all individual orthopedic subspecialties. Temporal trends were tested using linear regression.

Furthermore, the opioid prescription rate was modeled using a Poisson regression.^11^ The model used year, surgeon sex, average patient comorbidity risk score, and average patient age to predict annual adjusted incidence rates of opioid prescriptions. Poisson models were fitted to both the overall orthopaedic cohort and the individual subspecialty cohorts. Estimates of annual adjusted incidence rate ratios for the models were used to investigate changes in opioid prescription rate over time while controlling for included variables. All statistics were conducted using R version 4.0.2 (R Foundation for Statistical Computation, Vienna, Austria). A *P*-value less than 0.05 was considered statistically significant.

## Results

### Study Cohort

In total, we analyzed 5,026,911 opioid claims prescribed to 2,661,762 Medicare Part D beneficiaries between January 1, 2014, and December 31, 2018 (Table 1). This included claims submitted by an average of 7,417 unique orthopaedic surgeons per year. The most recent study cohort (as of December 31, 2018) comprised 7,306 surgeons, with the most common specialties being Sports Medicine (1,945; 26.6%), Hand Surgery (1,820; 24.9%), Spine Surgery (1,430; 19.6%), and Adult Reconstruction (1,035; 14.2%). Female orthopaedic surgeons comprised 6.0% of the study cohort, whereas the proportion of female surgeons across subspecialties ranged from 12.1% of hand surgeons to 1.4% of spine surgeons. The average mean patient age (SD) for subspecialty surgeons ranged from 68.38 (3.47) among orthopaedic trauma surgeons to 71.24 (2.36) among adult reconstruction surgeons. In addition, the hierarchical condition category risk score (SD) ranged from 1.05 (0.23) among sports medicine surgeons to 1.46 (0.40) among orthopaedic trauma surgeons (Table 1).

**Table 1:** Cohort Demographics by Year of Study and Orthopaedic Subspecialty

Year	2014	2015	2016	2017	2018
Overall, total claim count	2,235,553	2,214,655	2,268,251	2,291,115	2,250,051
Overall, opioid claim count (% of total)	1,097,065 (49.07)	1,042,574 (47.08)	1,034,121 (45.59)	953,980 (41.64)	899,171 (39.96)
Total no. of Medicare Part D patients	845,367	886,329	917,574	932,261	938,183
Total no. of Medicare Part D patients receiving opioid prescriptions (% of total)	523,163 (61.89)	543,156 (61.28)	548,555 (59.78)	536,651 (57.56)	510,237 (54.39)
Total no. of orthopaedic surgeons	N = 7,407	N = 7,480	N = 7,478	N = 7,416	N = 7,306
Subspecialty, no. (% of total)					
Adult Reconstruction	1,023 (13.8)	1,040 (13.9)	1,046 (14.0)	1,048 (14.1)	1,035 (14.2)
Foot and Ankle	572 (7.7)	570 (7.6)	582 (7.8)	573 (7.7)	590 (8.1)
Hand Surgery	1,823 (24.6)	1,842 (24.6)	1,848 (24.7)	1,855 (25.0)	1,820 (24.9)
Ortho Trauma	476 (6.4)	496 (6.6)	493 (6.6)	508 (6.9)	486 (6.7)
Spine Surgery	1,484 (20.0)	1,491 (19.9)	1,472 (19.7)	1,450 (19.6)	1,430 (19.6)
Sports Medicine	2,029 (27.4)	2,041 (27.2)	2,037 (27.2)	1,982 (26.7)	1,945 (26.6)
Female, no. (% within specialty)					
Adult Reconstruction	25 (2.4)	27 (2.6)	27 (2.6)	23 (2.2)	25 (2.4)
Foot and Ankle	62 (10.8)	62 (10.9)	62 (10.7)	66 (11.5)	69 (11.7)
Hand Surgery	206 (11.3)	213 (11.6)	216 (11.7)	217 (11.7)	220 (12.1)
Ortho Trauma	30 (6.3)	34 (6.9)	33 (6.7)	34 (6.7)	36 (7.4)
Spine Surgery	21 (1.4)	22 (1.5)	23 (1.6)	20 (1.4)	20 (1.4)
Sports Medicine	74 (3.6)	75 (3.7)	75 (3.7)	66 (3.3)	72 (3.7)
Medicare patient age, mean (SD)					
Adult Reconstruction	70.15 (2.84)	70.30 (2.79)	70.67 (2.58)	70.97 (2.52)	71.24 (2.36)
Foot and Ankle	67.72 (3.07)	67.81 (3.10)	68.17 (2.85)	68.55 (2.91)	69.00 (2.63)
Hand Surgery	69.16 (3.07)	69.35 (2.96)	69.63 (2.73)	69.91 (2.60)	70.18 (2.47)
Ortho Trauma	66.95 (4.09)	67.25 (4.09)	67.81 (4.06)	68.38 (3.55)	68.80 (3.47)
Spine Surgery	68.94 (3.38)	69.18 (3.30)	69.49 (3.10)	69.80 (2.96)	70.25 (2.86)
Sports Medicine	69.05 (3.14)	69.29 (2.96)	69.44 (2.93)	69.70 (2.82)	69.96 (2.62)
Medicare patient risk score, mean (SD)					
Adult Reconstruction	1.07 (0.24)	1.11 (0.23)	1.12 (0.24)	1.11 (0.24)	1.12 (0.23)
Foot and Ankle	1.17 (0.27)	1.23 (0.29)	1.21 (0.30)	1.21 (0.28)	1.20 (0.29)
Hand Surgery	1.07 (0.21)	1.16 (0.25)	1.15 (0.24)	1.16 (0.24)	1.16 (0.26)
Ortho Trauma	1.36 (0.35)	1.41 (0.36)	1.44 (0.39)	1.46 (0.37)	1.46 (0.40)
Spine Surgery	1.11 (0.21)	1.17 (0.21)	1.18 (0.21)	1.18 (0.22)	1.19 (0.22)
Sports Medicine	1.00 (0.22)	1.06 (0.23)	1.06 (0.23)	1.06 (0.23)	1.05 (0.23)

### Prescription Rate

**Figure 1:**
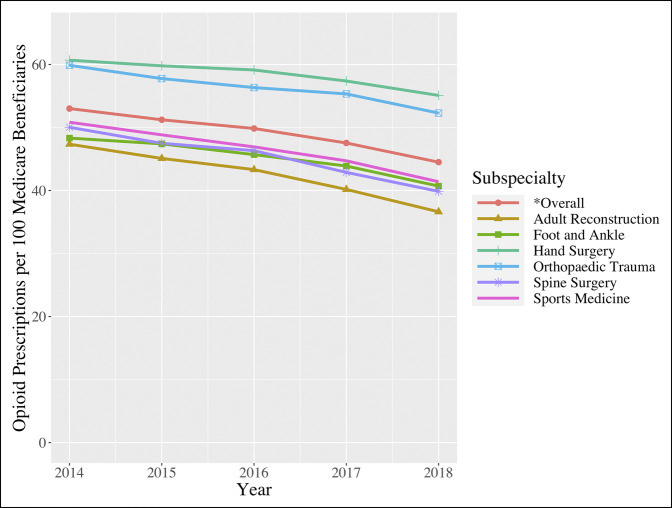
Line graph showing opioid prescriptions per 100 Medicare beneficiaries (2014 to 2018).

**Figure 2:**
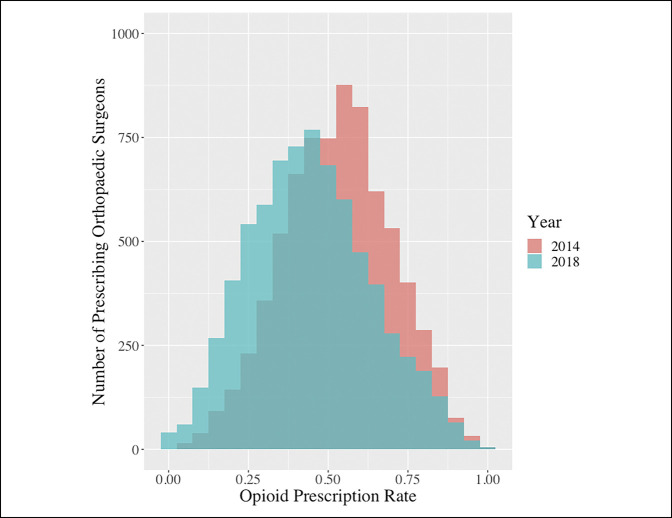
Histogram demonstrating the distribution of opioid prescription rates among included orthopaedic surgeons.

Among all orthopaedic surgeons, the rate of opioid prescription claims per 100 Medicare beneficiaries significantly decreased from 52.99 (95% CI, 52.60 to 53.37) in 2014 to 44.50 (44.50 to 44.93) in 2018 (*P* = 0.002) (Figure 1). When evaluating prescription rate distribution, rates in 2014 appeared normally distributed, with 57.18% of providers prescribing over 50 claims per 100 beneficiaries. Conversely, the distribution of prescription rates in 2018 was rightly skewed, with only 37.49% of orthopaedic surgeons prescribing over a rate of 50.00 (Figure 2).

Statistically significant decreases in opioid prescription rates were similarly observed for each orthopaedic subspecialty (Figure 1; Table 2). The largest decreases were observed among adult reconstruction surgeons (47.35 [46.37 to 48.32] in 2014 to 36.62 [35.54 to 37.70] in 2018; *P* = 0.001) and spine surgeons (50.03 [49.27 to 50.8] in 2014 to 39.86 [39.01 to 40.71] in 2018; *P* = 0.001). The smallest decrease in opioid prescription rates was observed among hand surgeons (60.68 [59.88 to 61.48] in 2014 to 55.09 [54.20 to 55.97] in 2018; *P* value = 0.006) (Table 2).

**Table 2:** Trends in Opioid Prescription Rate per 100 Medicare Beneficiaries by Orthopaedic Subspecialty

Year	Overall	Adult Reconstruction	Foot and Ankle	Hand Surgery	Orthopaedic Trauma	Spine Surgery	Sports Medicine
2014	52.99 (52.60-53.37)	47.35 (46.37-48.32)	48.32 (47.07-49.57)	60.68 (59.88-61.48)	59.86 (58.34-61.39)	50.03 (49.27-50.8)	50.83 (50.12-51.54)
2015	51.22 (50.83-51.62)	45.09 (44.07-46.11)	47.39 (46.09-48.7)	59.78 (58.99-60.58)	57.76 (56.2-59.32)	47.49 (46.71-48.27)	48.83 (48.11-49.55)
2016	49.84 (49.43-50.25)	43.31 (42.27-44.36)	45.69 (44.44-46.95)	59.13 (58.31-59.94)	56.33 (54.75-57.92)	46.32 (45.52-47.12)	46.92 (46.2-47.65)
2017	47.54 (47.12-47.96)	40.17 (39.13-41.21)	43.88 (42.58-45.19)	57.37 (56.54-58.2)	55.33 (53.73-56.92)	42.88 (42.06-43.69)	44.71 (43.97-45.45)
2018	44.50 (44.06-44.93)	36.62 (35.54-37.70)	40.70 (39.36-42.03)	55.09 (54.20-55.97)	52.29 (50.68-53.90)	39.86 (39.01-40.71)	41.39 (40.63-42.15)
*P* value	0.002	0.001	0.004	0.006	0.002	0.001	<0.001

All values expressed as the percent (%) of total Part D claims represented by opioid claims (95% CI).

### Opioids Claims Per Patient

**Figure 3:**
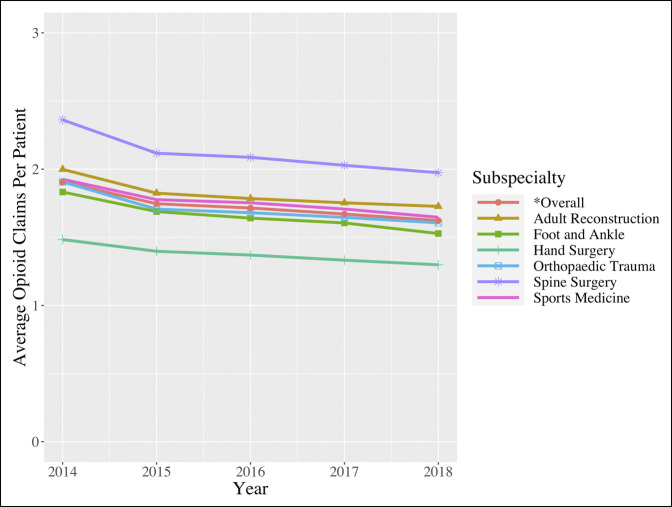
Line graph showing the average number of opioid claims per patient (2014 to 2018).

For all orthopaedic surgeons, the average number of opioid claims per patient also decreased significantly from 1.906 (1.888, 1.923) in 2014 to 1.623 (1.607, 1.638) in 2018 (*P* = 0.017) (Figure 3). Similar significant decreases were demonstrated when segregating for each subspecialty (all *P* values < 0.05) (Figure 3; Table 3). The largest decrease was seen for spine surgeons from 2.362 (2.337 to 2.387) claims per beneficiary in 2014 to 1.974 (1.953 to 1.996) (*P* = 0.029).

**Table 3:** Trends in Opioid Claims per Patient by Orthopaedic Subspecialty

Year	Overall	Adult Reconstruction	Foot and Ankle	Hand Surgery	Orthopaedic Trauma	Spine Surgery	Sports Medicine
2014	1.906 (1.888-1.924)	1.998 (1.984-2.013)	1.832 (1.817-1.848)	1.483 (1.473-1.493)	1.906 (1.892-1.921)	2.362 (2.337-2.387)	1.926 (1.911-1.941)
2015	1.746 (1.731-1.761)	1.824 (1.812-1.836)	1.689 (1.675-1.702)	1.397 (1.39-1.405)	1.707 (1.695-1.718)	2.117 (2.095-2.139)	1.776 (1.763-1.789)
2016	1.715 (1.699-1.731)	1.785 (1.773-1.796)	1.641 (1.628-1.654)	1.37 (1.362-1.377)	1.68 (1.669-1.692)	2.087 (2.064-2.109)	1.753 (1.739-1.768)
2017	1.671 (1.655-1.686)	1.753 (1.742-1.765)	1.605 (1.592-1.619)	1.332 (1.325-1.34)	1.646 (1.635-1.658)	2.028 (2.006-2.05)	1.707 (1.693-1.722)
2018	1.623 (1.607-1.638)	1.727 (1.715-1.738)	1.528 (1.515-1.541)	1.298 (1.291-1.306)	1.606 (1.595-1.618)	1.974 (1.953-1.996)	1.647 (1.631-1.663)
*P* value	0.017	0.036	0.007	0.005	0.039	0.029	0.013

All values expressed as number of opioid claims divided by the total number of Medicare Part D beneficiaries (95% CI).

### Days Per Opioid Claim

**Figure 4:**
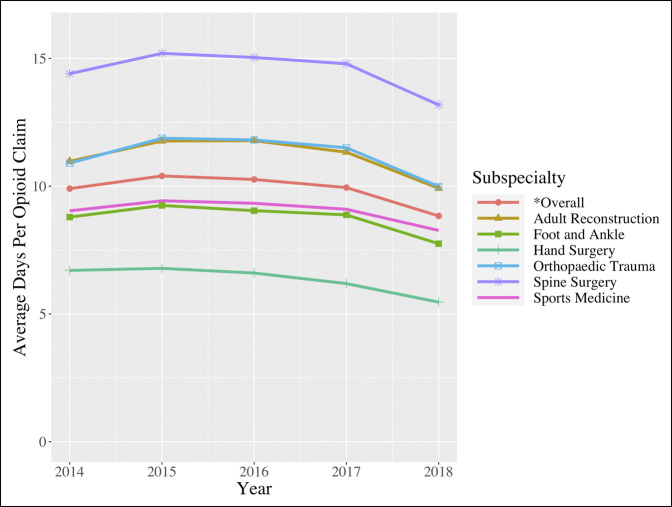
Line graph showing the average days of prescription per opioid claim (2014 to 2018).

When evaluating all orthopaedic surgeons, the number of days per opioid claim remained relatively stable between 2014 (9.906 [9.793 to 10.019]) and 2018 (8.835 [8.727 to 8.943]) (*P* = 0.219) (Figure 4). A similar nonsignificant trend was observed for included orthopaedic subspecialties, with the exception of hand surgery (Figure 4; Table 4). Specifically, hand surgeons reduced the number of days per claim from 6.704 (6.637 to 6.771) in 2014 to 5.463 (5.402 to 5.524) in 2018 (*P* = 0.042).

**Table 4:** Trends in Days per Opioid Claim by Orthopaedic Subspecialty

Year	Overall	Adult Reconstruction	Foot and Ankle	Hand Surgery	Orthopaedic Trauma	Spine Surgery	Sports Medicine
2014	9.906 (9.793-10.019)	10.976 (10.882-11.071)	8.791 (8.717-8.864)	6.704 (6.637-6.771)	10.9 (10.801-11.000)	14.402 (14.268-14.536)	9.036 (8.952-9.12)
2015	10.402 (10.283-10.520)	11.767 (11.667-11.868)	9.247 (9.171-9.323)	6.785 (6.715-6.856)	11.878 (11.773-11.983)	15.2 (15.066-15.334)	9.429 (9.342-9.516)
2016	10.264 (10.146-10.381)	11.782 (11.679-11.883)	9.042 (8.965-9.120)	6.601 (6.532-6.671)	11.812 (11.708-11.915)	15.038 (14.905-15.171)	9.331 (9.246-9.417)
2017	9.946 (9.830-10.063)	11.330 (11.233-11.428)	8.877 (8.8-8.954)	6.188 (6.121-6.255)	11.505 (11.403-11.608)	14.791 (14.659-14.923)	9.097 (9.014-9.18)
2018	8.835 (8.727-8.943)	9.916 (9.830-10.001)	7.749 (7.679-7.819)	5.463 (5.402-5.524)	9.991 (9.901-10.081)	13.182 (13.049-13.314)	8.269 (8.191-8.347)
*P*-value	0.219	0.363	0.219	0.042	0.458	0.328	0.238

All values expressed as the total day supply assigned to opioid claims divided by total opioid claims (95% CI).

### Poisson Regression

When adjusting for potentially confounding factors, the opioid prescription rate among all orthopaedic surgeons decreased significantly between 2014 and 2018 (annual adjusted incidence rate ratio = 0.833; 95% CI, 0.830 to 0.835; *P* < 0.05). Significant decreases in prescription rate were additionally demonstrated when evaluating each subspecialty independently (all *P* values < 0.05) (Table 5). As observed in the unadjusted analysis, adult reconstruction surgeons (0.784 [0.779 to 0.790]; *P* < 0.05) and spine surgeons (0.804 [0.800 to 0.809]; *P* < 0.05) saw the greatest decrease in opioid prescription rates over the study period, whereas hand surgeons saw the most modest decrease (0.946 [0.94 to 0.953]; *P* < 0.05) (Table 5).

**Table 5:** Poisson Regression Results for Adjusted Opioid Prescription Rate Ratios by Orthopaedic Subspecialty

aIRR (95% CI)	Overall	Adult Reconstruction	Foot and Ankle	Hand Surgery	Orthopaedic Trauma	Spine Surgery	Sports Medicine
Female	0.998 (0.993-1.003)	0.806 (0.791-0.821)	0.922 (0.909-0.936)	0.969 (0.962-0.976)	1.073 (1.050-1.096)	0.917 (0.905-0.930)	0.915 (0.905-0.926)
Patient risk score	1.057 (1.053-1.062)	0.996 (0.985-1.006)	1.019 (1.004-1.034)	0.795 (0.786-0.803)	1.175 (1.159-1.191)	1.171 (1.161-1.182)	1.069 (1.060-1.078)
Average patient age	0.976 (0.976-0.977)	0.967 (0.966-0.968)	0.966 (0.965-0.968)	0.974 (0.973-0.974)	0.984 (0.983-0.985)	0.986 (0.985-0.986)	0.974 (0.973-0.974)
Year							
2014	Reference	—	—	—	—	—	—
2015	0.960 (0.958-0.963)	0.947 (0.941-0.953)	0.980 (0.969-0.992)	1.012 (1.006-1.019)	0.95 (0.939-0.962)	0.943 (0.938-0.948)	0.961 (0.956-0.966)
2016	0.936 (0.933-0.938)	0.918 (0.912-0.923)	0.961 (0.949-0.972)	1.007 (1.001-1.014)	0.928 (0.917-0.940)	0.915 (0.910-0.920)	0.928 (0.923-0.933)
2017	0.888 (0.886-0.890)	0.859 (0.854-0.865)	0.935 (0.924-0.946)	0.979 (0.973-0.985)	0.89 (0.879-0.901)	0.855 (0.850-0.859)	0.883 (0.878-0.888)
2018	0.833 (0.83-0.835)	0.784 (0.779-0.79)	0.870 (0.859-0.880)	0.946 (0.94-0.953)	0.859 (0.848-0.870)	0.804 (0.800-0.809)	0.819 (0.814-0.824)

aIRR = adjusted incidence rate ratios

## Discussion

In response to the ongoing opioid epidemic, the orthopaedic community—one of the larger providers of narcotic prescriptions—has sought methods of curbing overprescription while safely and effectively managing patients' perioperative pain. Although various changes in legislation, institutional guidelines, and pain management techniques have emerged in recent years, there is limited information regarding how this has influenced the prescribing practices of orthopaedic surgeons across the nation. Therefore, our analysis aimed to provide a comprehensive, nationwide analysis of opioid prescription habits among orthopaedic surgeons caring for Medicare Part D patients. Although there were no substantial changes in the days per opioid claim over this study period, our analysis demonstrated statistically significant decreases in opioid prescription rates and the number of claims per beneficiary for all analyzed subspecialties. These findings were similarly demonstrated while controlling for various prescriber and patient characteristics.

Although the present analysis demonstrated statistically significant reductions in opioid prescriptions, it remains unclear whether these decreases can be considered clinically significant. According to a recent report by the Opioid Task Force of the American Medical Association, there was a 37.1% decrease in opioid prescriptions from 244.5 million in 2014 to 153.7 million in 2019.^12^ Our analysis demonstrated a more modest reduction of approximately 18%. However, this still translated to a reduction of 8.49 claims per 100 beneficiaries and a decrease in total opioid claims of 197,894. Therefore, although our analysis suggests that orthopaedic surgeons have meaningfully reduced opioid prescriptions over recent years to reduce the harmful effects of these medications, our findings additionally serve to encourage further reduction by both the AAOS and the wider orthopaedic community to ensure clinically notable change.

Multiple analyses have reported similar contemporary decreases in opioid prescriptions across orthopaedic subspecialties.^11,13,14^ Flanagan et al^13^ recently evaluated changes in opioid prescribing between 2012 and 2017 for orthopaedic trauma patients. After matching patients from each year by multiple variables, such as comorbidity burden and fracture location, the authors found significantly decreased total prescribed morphine milligram equivalents (MMEs) (1,110 versus 1,680 mg; *P* = 0.001) and refill amounts (766 versus 1,140 mg; *P* = 0.037) among the 2017 cohort compared with patients in 2012.^13^ Using a large claims database, Harris et al demonstrated a significant reduction in the number of patients receiving ≥90 MMEs after anterior cervical disectomy and fusion between 2010 and 2015 (1,811 [48%] versus 1,278 [43%]; *P* < 0.001).^11^ Goldman et al^14^ similarly reported a 27% decrease in oral MMEs when comparing patients undergoing primary total hip arthroplasty between 2014 and 2018. Our analysis further supports these findings by demonstrating statistically significant reductions in opioid prescriptions across a larger, national sample of orthopaedic surgeons.

Although there are various factors that likely have contributed to the decrease in opioid claims and prescription rates demonstrated in our analysis, federal and state legislation aimed at reducing the overprescription of opioids is considered one of the most influential.^15,16^ This has been reflected across the orthopaedic literature, with multiple studies demonstrating significant reductions in opioid prescriptions after the implementation of these laws.^17,18^ Glogovac et al reported significant reductions in both the overall number of opioid pills (49.7 versus 36.2; *P* < 0.001) and average MME per prescription (382.1 versus 275.2 mg; *P* = 0.016) when comparing patients undergoing surgical management of ankle fractures before and after Ohio implemented an Opioid Prescriber Law.^17^ After the passage of the Strengthen Opioid Misuse Prevention Act of 2017 (STOP Act) in North Carolina, Aran et al^18^ found a 35% decrease (27,374 mg; 95% CI, 13,226 to 41,523 mg; *P* = 0.0003) in total MMEs prescribed by orthopaedic surgeons at their institution. Coupled with the findings of the present analysis, these results should further encourage lawmakers to advocate for the widespread passage and implementation of similar prescription-limiting legislation. For states in which these have yet to be enacted, individual healthcare institutions should consider implementing similar protocols to further help combat the current opioid crisis.^19,20^

Our findings may also reflect an increased shift toward alternative methods of pain management for orthopaedic patients. Specifically, the implementation of multimodal and multidisciplinary pain management protocols has become increasingly popular, given their demonstrated effectiveness at reducing opioid consumption across orthopaedic subspecialties.^21-23^ Notably, Moutzouros et al^21^ found that 45% of patients undergoing sports procedures did not require breakthrough opioid analgesics when a multimodal protocol was used. Similarly, a recent analysis by Elkassabany et al^22^ found significantly higher quality of recovery scores at all time frames (*P*-values < 0.05) despite lower total MME consumption (111.2 versus 76.8 mg; *P* < 0.05) among shoulder arthroscopy patients in their multimodal pain management cohort compared with matched controls. Although continued research is needed to evaluate the efficacy of these protocols following more invasive orthopaedic procedures, providers should continue to consider methods of pain control that do not include opioid medications.

The authors acknowledge that despite the positive findings reported in our analysis, significant work remains in the orthopaedic community's push against opioid overprescription and abuse.^3^ A cornerstone of this effort relates to continued surgeon education regarding safe prescription practices and how to educate patients regarding the harms of opioid medications and misuse.^24,25,26,27^ These education programs have resulted in significant reductions in both the amount of opioids prescribed among orthopaedic surgeons and the opioids used by patients.^24,28-30^ Notably, Stepan et al^30^ reported significant decreases in the number of pills and total MMEs prescribed for sports (all *P*-values < 0.001) and hand (all *P*-values < 0.001) procedures after the implementation of an opioid prescribing education program at their institution. Similarly, there continues to be a push for uniform prescription guidelines, given inconsistent prescribing practices reported among providers.^31,32,33,34^ Although the development of consistent guidelines is challenged by wide variations in opioid consumption reported in the current literature,^35,36^ the implementation of institution-specific prescription protocols has resulted in notable decrease in excessive prescription practices.^37,38^ Therefore, continued research and development of these standardized protocols should be at the forefront of future efforts aimed at curbing the opioid epidemic.

The findings of our analysis must be considered in light of its limitations. We were unable to convert opioid claims into MMEs given limitations in the variables provided by the Medicare Part D PUF. Specifically, we were unable to control the type of opioid medication prescribed or the number of tablets given in each prescription. However, the conclusion that our analysis has drawn is more binary in nature because it is looking at reductions in the active number of opioid prescriptions rather than the specific amount prescribed per patient. Previous high-impact studies have used similar methodology when evaluating changes in opioid prescription rates without converting to MMEs.^12,15,39^ As many newly developed pain management regimens are aimed at eliminating opioid prescriptions entirely,^21,40^ rather than reducing the dose per prescription, evaluating how the number of opioid prescriptions among orthopaedic surgeons has decreased still provides important information to providers and policymakers working on reducing our effect on the opioid epidemic. In addition, we are unable to evaluate the accuracy of the taxonomy codes assigned to orthopaedic surgeons in the NPI registry. However, these codes are self-reported by healthcare providers when applying for their unique NPI and, therefore, likely can be considered accurate. We were unable to evaluate how prescription rates have changed after the implementation of various legislation in each state. Specifically, for the states that have implemented related legislation, there was large variability in the years that these laws were released as well as in the specific goals these laws aimed to achieve. Only approximately 70% of Medicare beneficiaries have Medicare Part D coverage, and therefore, our findings do not represent all prescriptions written for this patient population. Similarly, we were unable to evaluate the prescription patterns of orthopaedic surgeons managing younger populations and/or those with alternative insurance coverage. However, given that we reported on an average of 7,417 surgeons a year and over 5.03 million opioid prescriptions, our findings likely remain generalizable. Furthermore, we were unable to evaluate causes behind prescription variation between subspecialties because we did not have information regarding case-volume for each prescriber, their decision-making process for pain control, or how efforts made by related professional societies have influenced these decisions. Similarly, we were unable to control for how the age of our patients affected prescription decisions. Notably, although opioids are recommended with caution in older populations based on the Beers Criteria, other forms of pain relief, such as nonsteroidal antiinflammatory drugs may be contraindicated as well. We additionally were unable to assess changes in prescriptions for more conservative pain control medications or concomitantly prescribed medications over our study period. Similarly, over-the-counter medications, such as nonsteroidal antiinflammatory drugs, were not captured in the Medicare Part D files. Despite these limitations, we still found statistically significant reductions in opioid prescription rates across all subspecialties between 2014 and 2018.

## Conclusion

Orthopaedic surgeons across subspecialties have markedly reduced their rates of opioid prescriptions over recent years. Although increased prescription-limiting legislation, alternative methods of pain control and prescriber reeducation regarding the correct quantity of opioids needed for postoperative pain relief have likely contributed to these reductions, ongoing research and efforts are needed to translate these reductions into clinically meaningful changes. While these findings are promising, notable work remains in the orthopaedic community's push against opioid overprescription and abuse. To continue this trend, the AAOS and the leadership of subspecialty societies should evaluate effective methods of educating patients and providers regarding the harmful effects of opioid analgesics, shared decision-making in pain control, and the optimal amount of pain medication needed following specific orthopaedic procedures.
